# Injury characteristics, outcome, and return to play after ankle sprains and Achilles tendon ruptures in tennis

**DOI:** 10.17159/2078-516X/2026/v38i1a22949

**Published:** 2026-01-15

**Authors:** P Kaiser, B Petry, K Genelin, S Singh, T Ellenbecker, T Kastenberger, RA Lindtner, A Keiler

**Affiliations:** 1 Department for Orthopaedics and Traumatology, Medical University of Innsbruck, Innsbruck, Austria; 2 Sportklinik Arlberg, St. Anton, Austria; 3 Sportclinic Zillertal, Mayrhofen, Austria; 4 Banner Sports Medicine & ATP Tour, Scottsdale, AZ, USA; 5 Medicent Innsbruck, Innsbruck, Austria

**Keywords:** return to sports, ankle, foot, Achilles, tennis, sprain

## Abstract

**Background:**

Ankle sprains and acute Achilles tendon ruptures are common injuries among recreational tennis players.

**Objectives:**

This study investigated the specific cause, return to play, and ability to play tennis after an ankle sprain and Achilles tendon rupture.

**Methods:**

Patients who sustained an ankle sprain (n=39) or an Achilles tendon rupture (n=7) while playing tennis were retrospectively evaluated, with a mean follow-up of 6.0 and 5.2 years, and follow-up rates of 80% and 78%, respectively.

**Results:**

Ankle sprain patients were younger (39 years), smaller and lighter (BMI males 24.3, females 22.5) than Achilles tendon rupture patients (49 years, BMI males 26.8). Ankle sprains occurred more often on clay (56% [CI 40–72%]) and Achilles tendon ruptures on carpet courts (57% [CI 18–90%]). The return-to-play rate for tennis was 90% in patients with ankle sprains after a mean of 3.6 months and 29% in patients with Achilles tendon ruptures (two patients), with returns occurring at 5 and 36 months. The return to any sport was 97% and 100%, respectively. Twenty-nine players (74%) with an ankle sprain reported no complaints while playing tennis, and 6 (15%) had some instability or slight pain. Twenty-one per cent of all ankle sprain patients experienced subsequent events (1–3), with a 10% recurrence rate after a first-time event and a 33% rate in patients with recurrent instability episodes.

**Conclusion:**

The return-to-play rate for tennis was high after an ankle sprain and low after an Achilles tendon rupture. The reason for not returning in recreational players was either fear of re-injury or preference for other sports, not because of injury-related disabling factors. Patients after Achilles tendon rupture who returned to play tennis did not have any functional problems, and patients after ankle sprain rarely experienced minor instability or pain.

Approximately 87 million people (1% of the world population) play tennis according to the ITF Global Tennis Report of 2019. The estimated injury risk is up to 3.49/1000 playing hours among recreational tennis players.^[1]^ Many different acute and chronic injuries can occur in tennis. Chronic injuries tend to affect the upper extremities and back, while acute injuries tend to affect the lower extremities.^[2]^ The most common injury in tennis players is an ankle sprain (AS).^[3]^ Another acute injury in tennis players is an Achilles tendon rupture (ATR).^[4]^ Characteristic lower-extremity movement patterns in tennis have been studied and characterised. It was shown that, on average, tennis players change direction 4 times (range: 1–15) per point, with most running distances of up to 4.5 m per movement cycle.^[5,6]^ During the course of a tennis match, over 300–500 bursts of energy occur, highlighting the explosive demands on the lower extremity during tennis play.^[7]^

The characteristics, outcomes and return to sports of both injuries are well reported in the literature. However, sport-specific reporting is rarely conducted in sports such as soccer, basketball, American football, or volleyball. To our knowledge, there are no studies on the tennis-specific characteristics, outcomes, and return-to-play of recreational tennis players who sustained an AS or a complete ATR while playing tennis.

Therefore, the purpose of this study was to analyse the epidemiology of the tennis players with an AS or ATR, the specific cause of these injuries in tennis, and the return to and ability to play tennis after the injury. This study can offer better guidance for future patients who will sustain an AS or ATR, and the collected data may help prevent such injuries in cases of modifiable risk factors.

## Methods

All patients who sustained an AS or ATR while playing tennis between 2013 and 2018 and were treated in our emergency department (university hospital/department for traumatology) were invited to take part in this retrospective follow-up investigation.

In the AS group, patients who sustained a minor grade 1 ankle injury (ankle twist without a positive anterior drawer test of the ankle/grade I injury) were excluded from this study because the joint was stable during the clinical examination. The inclusion criteria for the AS group were pain, swelling, ecchymosis, and a positive anterior drawer test, with a diagnosis of ankle sprain documented in the medical chart (grade II or III). In the ATR group, patients who sustained an Achilles tendon strain or partial rupture were excluded. The inclusion criteria for the ATR group were a complete Achilles tendon rupture, diagnosed both clinically and by ultrasound. Thus, 49 patients with AS and 9 patients with total ATR were eligible to participate. Overall, 8 patients were unreachable, and 4 patients were unwilling to participate. Thus, 39 patients with an AS (women = 16; mean age 39 ± 16 years; range 15–70 years on the injury date) and 7 patients (all male; 49 ± 6 years; range 37–57 years on the injury date) with an ATR could be included. The local ethical review board approved this retrospective study. Informed consent was obtained from all participants.

All participants were required to complete a standardised questionnaire. The questionnaire was partly self-administered and partly conducted by an investigator. All assessments were carried out by the same investigator. Patients’ height (in cm), weight (in kg), BMI, dominant arm, dominant leg (defined as the leg which would kick a football), experience in tennis play (in years), playing ability (0–5; 0 = novice/5 = professional), ITN (International Tennis Number) ranking, and frequency of play (daily, several times per week, once per week, once every two weeks, once per month, once per year, first time) were recorded.

Injury characteristics included the date of the injury, the season of the year (spring, summer, fall, winter), playing singles or doubles, occurrence while playing (during a match or during training and in the beginning, middle or end of the play), the court surface (clay, carpet, hard, various [artificial grass, rubber, synthetic, red court]) and the side of the injury.

The patients were asked if they had warmed up, if they had any prior complaints in the lower extremity before the injury, and if they suffered from a recurrent ankle sprain or if it was a first-time event.

The exact tennis-specific moment of injury was recorded, as were the patient’s specific remarks regarding their perception of the cause of the injury. The treatment was distinguished between conservative and surgical therapy.

The patients were asked if they returned to playing tennis after recovering from the injury, if they participated in any other sport after the injury, when they started playing tennis again, and, if applicable, why they did not continue playing tennis.

Lastly, the treatment outcome was assessed. Patients were asked to report any complaints or pain (VAS) after the injury, whether they wore any orthosis while playing tennis, and if they encountered any problems during tennis play, as per the questionnaire by Maquirriain.^[8]^ The Foot and Ankle Ability Measure (FAAM) score was evaluated for both AS and ATR, along with self-reported function levels during sports (expressed as a percentage) and current functional status graded as normal, nearly normal, abnormal, or severely abnormal. Additionally, the Achilles Tendon Total Rupture Score and the Leppilahti Score were assessed for ATR.

All data are presented using descriptive statistics calculated by Microsoft Excel (Version 2016, Redmond, Washington, USA). Comparative statistical tests were not used due to the small sample size in the ATR group. The Clopper-Pearson Interval was calculated for the confidence intervals of the frequencies of AS and ATR on the playing surface.

## Results

The mean follow-up was 6.0 ± 1.9 years (3.0–8.8 years) for AS and 5.2 ± 1.8 years (2.6–7.3 years) for ATR, with a follow-up rate of 80% for AS and 78% for ATR.

Patient characteristics are shown in Table 1. Two patients with AS played nearly daily at the time of injury, 22 patients played several times per week, 10 played once per week, one played once per week, one played once every two weeks, one played once per month, two played once per year, and one player played for the first time in his life. Three players with an ATR played several times a week, two played once a week, one played once a month, and one played for the first time in their life

Twenty players with an AS had no ITN ranking and the rest had a mean ranking of 6.8 ± 1.9 (3.5–9.7). No player with an ATR had an ITN ranking.

During the outdoor season, 79% of all AS (spring n=11; summer n=15; fall n=6; winter n=6) and 86% of all ATR (summer n=5; fall n=1; winter n=1) occurred. Ankle sprains occurred more often on clay (56% [CI 40–72%]) and Achilles tendon ruptures on carpet courts (57% [CI 18–90%]). Injury characteristics are shown in Table 2.

All but one player with an ATR reported being subjectively warmed up, compared to all but five players with an AS (three did not recall).

Ten patients with AS reported subjective ankle joint instability before the injury, while two reported minor discomfort and pain (VAS 1–2) on loading. The specific AS was a first-time event for 21 players (54%) and a recurrent event for 18 players (46%; 1–6 times before this event to varying extents, mainly low-grade sprains). Only one player with an ATR had prior complaints due to a posterior cruciate ligament injury. No one suffered from pain.

Conservative therapy was applied for all AS, and none needed surgical treatment until the follow-up. The treating physician told all patients to wear an ankle brace for 6 weeks after the injury, day and night. Additionally, patients were encouraged to attend physical therapy to restore function and proprioception. Six ATR patients underwent surgical treatment, and one was treated conservatively.

All surgically treated patients underwent an open Achilles tendon repair using a Bunnell suture technique, followed by a strict functional rehabilitation protocol with full weight-bearing in a cast or boot with 20 plantar flexion immediately after surgery. Patients were immobilised for 6 weeks, and plantar flexion was successively reduced every 2 weeks, ending in a neutral position after 4 weeks. The conservatively treated patients underwent a similar rehabilitation protocol; however, it began with full plantar flexion at 30°. The ankle was immobilised for 8 weeks, with progressive reduction of plantar flexion every 2 weeks, reaching neutral position after 6 weeks. Conservatively, as well as surgically, treated ATR patients started physical therapy with an active range of motion after the immobilisation period.

**Fig. 1:**
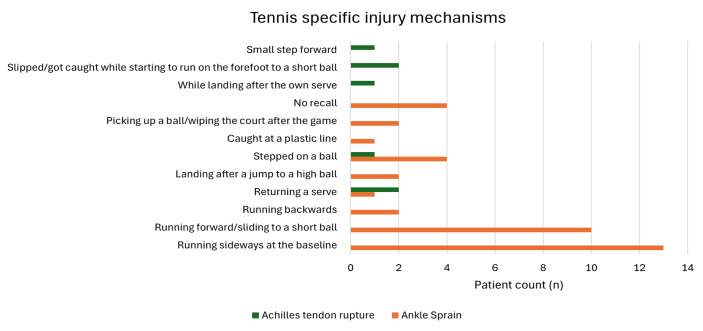
Tennis specific injury mechanisms (n=46)

The mechanisms of injury are presented in Figure 1. ATR usually occurred with a sudden push-off with the forefoot. A wet court was reported as the cause of an injury in three AS patients, and excessive clay on the court was reported as the cause in another three patients. One patient who sustained an ATR was a forefoot marathon runner for many years with a chronic tendon overload and a 10 km run the day before. Eleven per cent of all patients (4 AS/1 ATR) stepped on a ball while playing.

The return-to-play rate for tennis was 90% (35/39) in AS patients, with a mean time to return of 3.6 ± 2.9 months (range, 1.0–12.0 months). However, the time of return was also dependent on the time of the year. If playing outdoors was still possible, the mean time was 2.2 ± 1.4 months (1.0–7.0 months); otherwise, players usually waited and played after the winter season ended. Additionally, the time to return to play was 4.4 ± 3.4 months (1.5–12 months) after a first event, compared with 2.9 ± 2.0 months (1.0–7.0 months) after a recurrent event. The return-to-play rate for tennis was 29% (2/7) in ATR patients, with returns occurring at 5 and 36 months. Excluding novice first-time players, the return rate was 40% (2/5). One player with an ATR who returned to play tennis is now only playing on carpet or grass, but not on clay, due to fear of sliding after sustaining the injury on clay. The return to any sport (RTS) was 100% in ATR and 97% in AS patients (mainly jogging, cycling, mountain biking, skiing, cross-country skiing, ice hockey, mountain hiking, Tabata/fitness training).

**Fig. 2:**
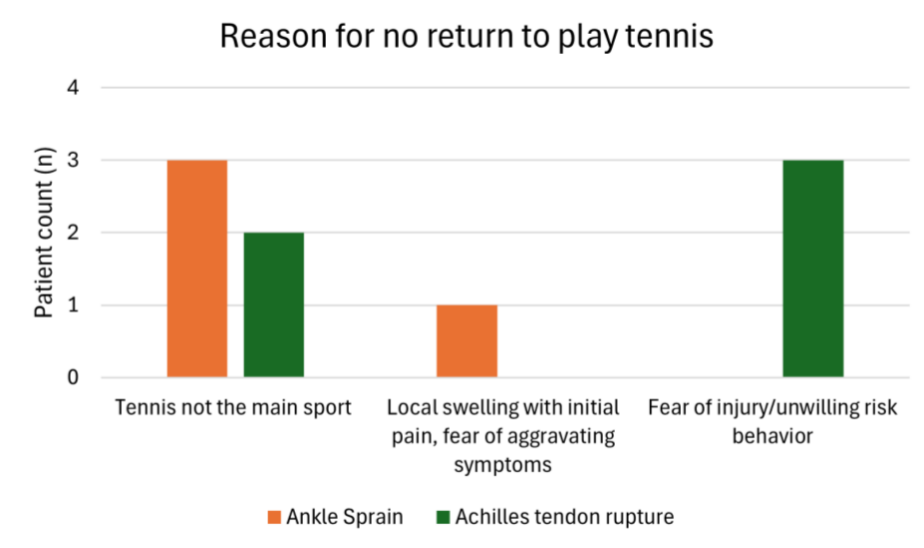
Reason for no return to play tennis

The patient (male, 49 years, BMI 29, FAAM Score 44, no pre-injury complaints), who did not return to any sport after an AS, had only a single ankle sprain event and no recurrence. The reason for not returning to play tennis is shown in Figure 2. Only two players had minor pain at rest (VAS 1) after an AS and 11 players had some pain while under load (VAS 2.0 ± 1.4 (1–5)). Twenty-eight players (72%) reported no pain. Twenty-four players (62%) had no complaints at all, while 7 players (18%) experienced occasional instability, and 8 players (21%) reported local tenderness. Only one player reported a pain level of 0 at rest and 4 under load after an ATR due to some Achilles tendon tension, and another one reported some initial tension and a feeling of a lower push-off force.

Thirty-one patients (79%) did not have a recurrent event after this specific AS, while 8 (21%) had further events (1–3 times). The recurrence rate after a first-time event was 10% (2/21). The recurrence rate in patients who sustained a recurrent event was 33% (6/18) (Table 3).

Pain was reported by seven patients (33%) with AS who only sustained a single event and by four patients (22%) who had recurrent events. The four patients with AS who did not return to tennis had a first-time event with no recurrence. Only patients with recurrent events reported some subjective instability, while all patients with a first-time event and no recurrence felt subjectively stable.

Twenty patients (51%) with an AS did not wear any external stabilisation while playing tennis after their initial healing phase of 6 weeks, while 15 players (28%) wore some external stabilisation (brace, orthosis or tape) for a period of another month up to two years after the 6 weeks healing time after the injury (Table 4). The reason for prolongation was either a subjective feeling of instability or fear of re-injury. One player with an ATR used customised shoe inlays from time to time, but no orthosis.

The FAAM Score was 95 ± 11% (44–100%) and the self-reported level of function during sport activities was 93 ± 10% (60–100%) in AS. It was 97 ± 7% (81–100%) and 93% ± 6% (85–100%) in ATR. Twenty-three patients (59%) with an AS reported to have a normal, 15 (38%) a nearly normal and one patient (3%) an abnormal current level of function of the ankle joint. Four patients (57%) with an ATR had a normal current level of ankle joint function, and 3 (43%) had a nearly normal level. The Achilles Tendon Total Rupture score was 99 ± 2% (96–100%), and the Leppilahti Score was 87 ± 16% (60–100%).

The ability to play tennis was very good after an AS or ATR, according to the questionnaire by Marquirrian.^[8]^ Twenty-nine players (74%) with an AS reported no complaints at all, while six (15%) had some instability issues or slight pain. Results were slightly worse for patients with recurrent AS than for those with a first event, especially when moving from baseline onward (mean 4.6–4.7 vs. 4.9–5.0). Specifically, some players had difficulty sprinting to one side of the court or the other after an ankle sprain. Patients also reported limitations in moving forward to reach a drop shot, moving forward after serving to volley, and stopping abruptly and turning direction. The players did not report pain, but rather a feeling of instability. A graded scale rating of 4 means "able to do it with some instability and no pain.” Both players with an ATR who returned to play tennis didn’t have any problems at all (graded scale rating of 5).

## Discussion

The tennis-playing patient population who sustained an AS and an ATR differed. Patients with an AS were smaller, lighter, and younger by approximately 10 years on average than patients with an ATR. Professional athletes who sustain an ATR seem to be younger.^[9]^ However, in the general population, a peak occurs around age 40–49, which is comparable to the patients’ ages in this cohort. This peak is likely due to a rising participation in recreational sports in middle-aged people. The Achilles tendon may be degenerated or not prepared in “weekend warriors” due to the “new” load.

Interestingly, only men sustained a complete ATR in this study. The reason may be because of the overall low number of patients, and probably because more men play tennis. Thus, there may be a potential bias because the male-to-female ratio of ATR in the general population is 2–12:1.^[10]^ However, if the finding that only men sustain a full ATR in tennis is true, there may be an intrinsic effect in tennis that explains why women are not at risk for this injury in this sport. Further research would be necessary to verify this finding. Some females sustained a partial (10–50%) ATR. Nevertheless, they were not included in this study because only complete ruptures were investigated.

The mechanism of injury differs between AS and ATR. Achilles tendon ruptures typically occur with a sudden forced plantar flexion moment developed by a strong calf contraction and often a simultaneous knee extension or a violent, potentially unexpected dorsiflexion force on a plantar flexed foot.

These mechanisms appear to be similar to the reported mechanisms of injury. An unexpected, forced dorsiflexion may occur while stepping on a ball. More often, a forced plantar flexion seems, however, to be the reason for rupture. Returning a serve with a sudden push-off moment is risky. Other forceful moments can include a sudden start and pushoff on a short ball, or the landing after a forceful serve, potentially with a straight knee joint, leading to over-tensioning of the tendon. Because injuries occurred frequently in individuals with a higher BMI and relatively novice players, one may conclude that these players, who have risk factors for Achilles tendon ruptures (increased age, higher BMI, Achilles tendinopathy, corticosteroid intake or previous injections, previous quinolone antibiotic intake, chronic tendon overload), should avoid these movements. This means they may be advised not to start running to a short ball in the beginning of their tennis “career” and they should not play competitive matches with multiple sudden returns of serves until their tendon are adequately trained and get used to the new load.

The injury mechanism in AS in tennis is mainly running and sliding to a short ball near the net or at the baseline. These mechanisms in recreational tennis players are similar to those of professionals in a retrospective video analysis. That study revealed that large, sudden inversion and internal rotation, but not plantar flexion, occur in tennis players during ankle sprains. This is different from basketball and volleyball because there are more horizontal and sideward movements in both medial and lateral directions, but fewer vertical jum-planding motions. Additionally, the foot is planted on the tennis court and cannot be further plantarflexed. If possible, ankle inversion should be avoided in tennis, and players should keep their centre of plantar pressure from shifting laterally to prevent ligament sprains.

Although uncommon, stepping on a ball can also lead to this injury. Interestingly, players stepping on a ball caused around 10% of injuries in both groups. This proportion seems high, given that stepping on a ball may be a rare event in tennis. Stepping on a ball may thus be an avoidable external risk factor for sustaining an AS or ATR while playing tennis, and players should strongly be advised to clear the court of any remaining tennis balls before recommencing play.

Neuromuscular training programs may improve foot position and ankle stability during sliding and tennis strokes. Although this training is effective in preventing recurrent sprains, it remains unclear whether the risk of a first-time event can also be reduced.

The recurrence rate after the specific AS event investigated in this study was 21%; however, only 10% of the patients with a first-time AS event sustained a recurrence. This is lower than previously reported, with a chronic ankle instability prevalence of 40% within one year after a first-time event in a prospective cohort.

Interestingly, none of the patients with a first event who wore the ankle orthosis for a more extended period than the recommended 6 weeks sustained a recurrence, in comparison to two patients who did not prolong wearing the orthosis and sustained a recurrence (Table 4). Any external ankle stabilisation, like non-rigid ankle bracing, taping or orthosis, can reduce the risk of first-time AS and recurrent events by up to 50–70%.^[11]^ Therefore, tennis players may be advised to wear an ankle fixation for an additional 6 to 12 months after the event.^[11]^

Similar to other reports, which identified the proportion of recurrent AS to range between 12–47%^[12]^, 46% of AS were recurrent, and these patients probably suffered from a chronic, unstable ankle joint. The fact that these patients suffered from chronic ankle instability can also be deduced from the fact that half (9/18) of these patients wore any external ankle stabilisation for a more extended period than only for 6 weeks.

Although the literature states that up to 70% of patients may experience complaints of the ankle joint, the symptoms in this cohort with an AS were rare and of minor relevance, with high functional scores and a high level of function. No patient in this study needed surgical treatment within a mean of 6 years. Yet the rate of patients with chronic ankle instability is high, and these patients may potentially need surgical treatment in the future because chronic ankle instability may lead to degenerative changes. However, the level of degenerative changes and future progression cannot be deduced in our cohort. The natural course needs to be awaited, and future investigations with this cohort may help to answer this question.

One very important finding of this study was the high return to play tennis rate after an AS (90%) and low rate after a total Achilles tendon rupture (29%) in recreational tennis players. While the main reason for not playing tennis anymore in AS is that tennis is not the individual’s primary sport, they still participate in other sports, with a return-to-sport rate of 97%. There was only one patient who did not perform any sport anymore because of a local intermittent swelling, pain and fear of aggravating symptoms after a single AS event. Whether the ankle joint limited him from participating in any sport or whether the sport itself was not crucial for this person remains unknown. In contrast, the return-to-sport rate was 100% after an ATR; however, the return to tennis was low (29%), primarily due to psychological factors. The persons did not report any relevant limitations due to their ankle joint or Achilles tendon, but they feared re-injury and were unwilling to take the risk while playing tennis.

The return-to-play tennis rate after an ATR appears to be lower than reported in the literature (61%–100%) for professional athletes in various sports.^[9,13]^ The return-to-play seemed dependent on the sport itself as soccer players are more likely to return than basketball, football and baseball players.^[9]^ Around 30% of all players will not return to play, and 25% will not be able to achieve their pre-injury performance, especially within the first year after injury, but will show some improvement within the second year. No data is available for professional or recreational tennis players. Grassi et al. reported on eleven soccer players from the Italian first division who all returned to professional soccer after their initial injury.^[13]^ This suggests that a healed Achilles tendon rupture can lead to a resilient structure in professional athletes. Therefore, playing tennis recreationally may be possible for nearly everyone after an ATR, given the biomechanical ankle properties and requirements of the sport. Among the two patients in this study who returned to playing tennis after an ATR, neither had limitations while playing and could perform all tennis strokes without complaint.

The high return-to-play (100%) also suggests that the ankle joint and the tendon are strong and resilient. Yet, many patients did not return to play tennis again. The reasons were mainly psychological. All these findings are similar to those of Peterson et al.^[14]^ and Slagers et al. investigations^[15]^ on psychological factors during the rehabilitation of ATR. The former investigators reported a similar low return-to-play rate of around 20–30% in recreational tennis/paddle tennis, basketball, soccer, and other sports. The main reasons were a shift of focus and fear of re-injury (65%). However, similar to our patients, 100% of patients returned to any sport. Often, people realise that their body does not function as it used to after turning 30, 40, or 50 years, and they change their priorities, which is also common among many other injuries, including knee, elbow, hip, and shoulder surgeries.^[14]^ Fear can be a significant psychologically limiting factor, as even one patient who was included in this series returned to play tennis but had fear and avoided playing on clay. Slagers et al.^[15]^ saw that fear for re-injury and fear of having to repeat rehabilitation are essential reasons for patients for not returning to the pre-injury sport. Although all participants returned to sports in our study, these were low-risk sports regarding re-rupture. Psychologically, they suffered from some kinesiophobia (fear of pain due to movement) regarding the return to play tennis again. Patients can show a low motivation level to return to play tennis and a change in priorities, which may potentially be unchangeable, however, also unimportant, as these patients participated in other sports which are beneficial with respect to overall healthy ageing. Some other patients may have low psychological readiness and confidence in their return to play tennis with a high level of kinesophobia despite a wish to play again. These patients may benefit from physical therapy and psychological interventions to enhance motivation, psychological readiness, and reduce kinesophobia. It is unclear whether the injury or rehabilitation was so traumatising for the patients that they are afraid of a re-injury or whether the treating physician scared the patients by explaining to them the high re-rupture rate, especially in stop-and-go sports. Patients with an ATR may benefit from a more accurate explanation regarding playing tennis.

AS showed a much higher return-to-play rate, likely due to lower injury severity. Therefore, these psychological effects are not particularly pronounced, except that players - especially those suffering from recurrent ankle sprains – may have some fear of recurrence. That’s why some players continue to wear external ankle stabilisation. Most players dispense the stabilisation once they feel the ankle is sufficiently stable again.

Because of the low severity of AS, the return-to-play is also a lot shorter. The return to play tennis after an AS showed a wide range of 1 to 12 months. The time of year played an important role because players who injured themselves around winter waited until spring to return to tennis. Additionally, players with a first-time event (mean 4.4 months) waited a mean of 1.5 months longer to return to play tennis than players with a recurrent event (mean 2.9 months). The time to return to play seems longer than reported in the literature, with a return to play within 7 to 15 days.^[16]^ Our study did not include grade I injuries, which may explain this difference, as higher-grade injuries take significantly longer to return to play.

The return-to-play after an ATR was delayed, due to the tendon’s extended healing time. The literature reports a wide range of mean return-to-play after an ATR (5.6 – 12.3 months). However, early return (< 3 months) is also reported in a few cases.^[9,13]^

Importantly, patients should not be encouraged to return to practice, play, or compete before 6 months after the injury, as most re-ruptures occur within 3 – 6 months of the initial injury, with an overall re-rupture rate ranging from 1.6% to 15%.^[9,10,17]^ There was no re-rupture seen in this cohort, neither while playing tennis nor while performing other sports. This is likely due to the low patient count and the low participation in stop-and-go sports.

Finally, AS occurred more often on clay courts, the dominant court surface in our area, whereas ATR occurred primarily on carpet. Whether this finding is accidental or specific cannot be determined because the overall number of players on each court surface is unknown. However, one can imagine that, on the one hand, players would rather try to slide on clay than on other court surfaces, leading to AS. On the other hand, a carpet surface may produce different ground reaction forces on the Achilles tendon compared to clay, potentially increasing the risk of an ATR. Other investigators did not find a difference in the overall injury prevalence among recreational players, regardless of playing surface. However, a higher prevalence was observed among players who played on multiple surfaces—a factor not investigated in this study.

Contrary to this, there was a higher count of incomplete matches among professional players, indicating match retirements due to injury, for women on Australian hardcourts and for men on USA hardcourts during the Grand Slam tournaments, and fewer injuries on grass.^[18]^ The relative risk for medical treatment during the ATP tour was lower on hard courts than on grass courts (RR 0.8). However, hard courts showed a higher relative risk than clay courts (RR 2.3).^[19]^ Other researchers have reported that the overall risk of injury appears lower on clay courts and that tennis players who have played their careers on clay have fewer knee problems than those who have played on hard courts.^[20]^ In summary, the optimal court surface for reducing injury risk in recreational players remains unknown.

The retrospective character of this study is an obvious limitation, as precise reporting was often based on potentially vague memories and recall. Additionally, the patient count, especially for ATR, was low, but the follow-up rate was high (80% and 78%). Objective measures of the range of motion, joint strength or functionality, and anatomical characteristics of the ankle and lower limb are missing and can be investigated in future research. Prospective study designs, including a large tennis-playing population with a homogeneous type of injury, should be conducted in the future to comprehend better playing performance and the return to tennis after each single injury.

## Conclusion

In recreational tennis players, the return-to-play rate after an ankle sprain during tennis is high (90%), compared with a lower rate after a complete Achilles tendon rupture (29%). However, the return-to-sport rate remains high after both injuries (97% and 100%, respectively). The main reasons for not playing tennis anymore were either that tennis was not the person’s main sport or that the patients feared re-injury and were unwilling to take the risk. Individuals who returned to play tennis after an Achilles tendon rupture did not appear to experience any functional problems during play. In contrast, patients who sustained an ankle sprain typically experienced minor instability or, in selected cases, pain while playing tennis.

## Figures and Tables

**Table 1:** Patient demographics (n=46)

	Ankle sprain (AS) (n=39)	Achilles tendon rupture (ATR) (n=7)
Height (males) (cm)	182 ± 6 (168–198)	186 ± 2 (182–188)
Height (females) (cm)	166 ± 4 (157–175)	n/a
Weight (males) (kg)	81 ± 11 (62–105)	93 ± 8 (82–104)
Weight (females) (kg)	62 ± 10 (44–85)	n/a
BMI (males) (kg/m^2^)	24.3 ± 2.6 (18.8–29.2)	26.8 ± 2.2 (23.7–29.4)
BMI (females) (kg/m^2^)	22.5 ± 3.5 (17.9–31.6)	n/a
Dominant arm*	34 right-handed/5 left-handed	6 right-handed/1 left-handed
Dominant leg*	34 right-footed/3 left-footed/2 both footed	6 right-footed/1 left-footed
Years of experience (y)	19 ± 14 (0–50)	11 ± 10 (0–30)
Playing strength (n)	0 (1); 1 (7); 2 (12); 3 (16); 4 (3)	0 (2); 1 (1); 2 (3); 3 (1)

Data shown as mean ± SD and (range).

* Racquet arm and limb were ipsilateral for 34 and contralateral in 3 patients with AS, and ipsilateral for all with ATR. BMI, body mass index.

**Table 2:** Injury demographics (n=46)

Match information		Ankle sprain (n=39)	Achilles tendon rupture (n=7)
Match type	Singles/doubles	27/12	5/2
Game type	Playing/match/training	10/21/6 (+ 2 after the game)	2/4/1
Time of injury	Beginning/middle/end of play	9/16/14	2/3/2
Court surface	Clay/carpet/hard/various* court surface	22/7/4/6	2/4/0/1
Injured side	Right/left	19 right/20 left	4 right/3 left

* various = artificial grass, rubber, synthetic, red court

**Table 3:** Patient count (%) who sustained a first-time ankle sprain (AS) and recurrent AS including further recurrence (n=39)

	No recurrence after event	Recurrence after event
First time AS	19 (90%)	2 (10%)
Recurrent AS	12 (67%)	6 (33%)

**Table 4:** Patient count (%) who wore any external stabilization longer than 6 weeks after the specific ankle sprain (AS) event in tennis (n=39)

	No recurrence after event	Recurrence after event
First time AS (n=21)	6/19 (31%)	0/2 (0%)
Recurrent AS (n=18)	4/12 (33%)	5/6 (83%)
